# Man as a Subject of the Reality of Health: a Personal Reflexion

**DOI:** 10.5935/1678-9741.20160009

**Published:** 2016

**Authors:** Alexandre Visconti Brick

**Affiliations:** Universidade de Brasília (UNB), Brasília, DF, Brazil and Programa de Pós-Graduação em Ciências da Saúde of the Faculdade de Medicina de São José do Rio Preto (FAMERP), São José do Rio Preto, SP, Brazil.

## PERSONAL REFLECTION

We have so much to learn. The world is as mysterious as challenging and therein lays the beauty and soul of curious researcher.

Leonardo Da Vinci affirmed that knowledge makes the soul younger and reduces the bitterness of elderly age. So when we awake, despite all the problems of modern society, we must reap wisdom and store softness for tomorrow.

The quest for knowledge is a source of joy it is the root of the findings and inexhaustible source of progress.

Examples of exciting love of knowledge changed the history of humanity. Leonardo Da Vinci was a proof of brain power for life. In addition to the master painter, he was an architect, mechanic, engineer, chemist, botanist, geologist and also developed several other activities. Maybe he was the first to study the human body dissecting corpses, prohibited action at that time and now so important to the learning of everybody of health area.

Surely science and technology have done wonders in many fields, but I agree that they did not stimulate goodness. When we remember figures such as Da Vinci and the Dalai Lama, we should take them as examples of great men who changed the world, because they were great observers, its own characteristic of great scientists.

What we want represents what is best into universities, it is a time of great curiosities, many questions, intense studies, inquiries and research and we can immortalize youth through knowledge and make smooth each new day (our and everybody's) to share the blessings of wisdom.

The great and immortal Pasteur has already stated: "Science and peace will triumph over ignorance and war. People will understand themselves not to destroy but to build." In ancient times it reflected a desire which still remains.

What is happening on the national scene is the existence of a defective medical education with needy universities, creating new schools, when in fact, we should think about removing some, because we cannot give them the least consistent conditions with its superior design. The demerit is not private medicine, because we still have illiteracy in Basic, Secondary and also in Degree education, intercommunicating in his weakness because its lack of a solid foundation.

It is very serious that students traverse of faltering way these various stages and come to the university, it should accept the brain of the nation. Must have a demanding and selective teaching, no prejudice on creed, color, sex or nationality.

Participating in enlightened meetings (politicians and others) that we hear proposals that every student that conclude the high school could raise degree education without entrance exam or defending the free profession without diplomas, another barbarity that only weakens universities looking leveling down.

University teachings in health in Brazil were initiated by the French school, which favored the patient as a whole. Today we see with rare exceptions it is a mercantilist teaching, demagogic and electioneering often sponsored by the agencies that should regulate medical practice.

For sure, what we all want, with USH (Unified Health System) or without USH, is a social medicine of great magnitude, covering the entire Brazilian population, preventive and curative medicine and that universities and university hospitals are the source of knowledge based on education , research and extension focused on the human individual.

"Where are the true sources of human dignity, freedom and democracy, but in the infinite sense before which everybody are equal?" Questioned by Pasteur in his prayer of thanks to Renan, who greeted him to join the French Academy. And the same Renan said: "Life is a moment between two eternities, the one that precedes the birth and that succeeds death".

Medicine and health are human activities toward humans. The clinical encounter is their central point in the diagnosis, the rest is addition, nothing more than people working tools taking care of people.

The increasing use of technology is welcome and signifies progress, but its use must be done judiciously, targeting benefits without risk to patients and effectively viable costs.

It discusses what should be modified or implemented first, finance, management, human resources or health care model.

I see no possibility of separating these sectors: if there is no funding, no models will implement, not improve efficiency and no human resources in quantity and quality needed.

The model approach to ensure health should be reviewed. The induction ducts order tests led people to demand the right such practices costly tests made by experts.

On the other hand promoting and preventive practices in relation to disorders health and sanitation policies should be prestigious, incorporate health professionals and integrated with each other.

There is no possible to treat strokes (human suffering and higher expenses) when not dedicate time and resources to the effective control of blood pressure. The same should be in relation to vector-borne diseases caused by lack of sanitation.

Do not discuss a poor medicine, but a rationalization of the global focus on the health of human, with simple steps that will have better and long-term effects on citizens, society and the quality of life for all^[1-4]^.

Some Facts to Ponder:

Federal Constitution - "Health is a right of everyone and a duty of the state" - Brilliant!

How to make? Who pays?

The reality is that over 50% of people who go to consultation are not carrying any organic disease; 50% of diseased, about half is sick because poor habits. In 90% of cases, the conversation between doctors and the patient is able to establish a diagnosis. Has anyone paid attention to the ad agencies that say "Health" and offering most modern ambulances, helicopters, next-generation devices and stay in ICU (Intensive Care Unit) for 365 days?

In public hospitals, large numbers of dependent patients of surgery to treat stomach cancer and cholecystitis are operated only at an advanced stage of their disease with obvious impacts on morbidity/mortality.

They found that the number of pharmacies in our neighborhood exceeds the real needs?

So how about to face this health frenzy in the private field with the health neglect in the public service?

"The state seriously injured and, stills, the Federal Constitution with impunity" (Luiz Roberto Londres)

We are producing first world advertising and pushing the poor to a sanitary underworld with consequent regression of health.

"The Education of a nation, according to what history teaches us, is one of the greatest weapons for health. How much more health, culture and wisdom have a nation, much more it will be happy" (Professor Mario Rigatto)
